# Effect of Annealing Temperature on the Mechanical and Corrosion Behavior of a Newly Developed Novel Lean Duplex Stainless Steel

**DOI:** 10.3390/ma7096604

**Published:** 2014-09-12

**Authors:** Yanjun Guo, Jincheng Hu, Jin Li, Laizhu Jiang, Tianwei Liu, Yanping Wu

**Affiliations:** 1 Department of Materials Science, Fudan University, No. 220, Handan Road, Shanghai 200433, China; E-Mail: 13110300027@fudan.edu.cn; 2 Research and Development Center, Baosteel Co., Ltd., No. 885, Fujin Road, Shanghai 201900, China; E-Mails: hujincheng@baosteel.com (J.H.); lzjiang@baosteel.com (L.J.); 3 Science and Technology on Surface Physics and Chemistry Laboratory, P.O. Box 718-35, Mianyang 621907, Sichuan Province, China; E-Mail: liutianwei@caep.cn; 4 China Academy of Engineering Physics, P.O. Box 919-71, Mianyang 621900, Sichuan Province, China; E-Mail: wuyanping@caep.cn

**Keywords:** lean duplex stainless steel, annealing temperature, mechanical properties, TRIP effect, pitting corrosion behavior, PREN, Volta potential

## Abstract

The effect of annealing temperature (1000–1150 °C) on the microstructure evolution, mechanical properties, and pitting corrosion behavior of a newly developed novel lean duplex stainless steel with 20.53Cr-3.45Mn-2.08Ni-0.17N-0.31Mo was studied by means of optical metallographic microscopy (OMM), scanning electron microscopy (SEM), magnetic force microscopy (MFM), scanning Kelvin probe force microscopy (SKPFM), energy dispersive X-ray spectroscopy (EDS), uniaxial tensile tests (UTT), and potentiostatic critical pitting temperature (CPT). The results showed that tensile and yield strength, as well as the pitting corrosion resistance, could be degraded with annealing temperature increasing from 1000 up to 1150 °C. Meanwhile, the elongation at break reached the maximum of 52.7% after annealing at 1050 °C due to the effect of martensite transformation induced plasticity (TRIP). The localized pitting attack preferentially occurred at ferrite phase, indicating that the ferrite phase had inferior pitting corrosion resistance as compared to the austenite phase. With increasing annealing temperature, the pitting resistance equivalent number (PREN) of ferrite phase dropped, while that of the austenite phase rose. Additionally, it was found that ferrite possessed a lower Volta potential than austenite phase. Moreover, the Volta potential difference between ferrite and austenite increased with the annealing temperature, which was well consistent with the difference of PREN.

## 1. Introduction

Duplex stainless steels (DSSs), strongly relying on a balanced two-phase microstructure of ferrite (α) and austenite (γ), have attractive mechanical and corrosion properties and are thus widely used in the chemical, petrochemical, nuclear, marine and paper industries [1,2,3,4]. In past years, the development of DSSs has followed two routes. On one side, great efforts have been made to improve the corrosion resistance of DSSs by increasing the content of chromium (Cr), molybdenum (Mo) and nitrogen (N), e.g., the DSS UNS S32750 with high mass fractions of 25%–27% Cr, 3%–4.5% Mo and 0.25%–0.28% N, which has been developed to meet with the requirement of good resistance and high strength in severe service environment [5,6,7]. On the other side, in order to conserve resources, “lean” route DSSs, e.g., UNS S32101 type with less nickel (Ni) but higher yield strength and better pitting resistance than standard austenitic grades, have been developed to meet the demand for grades with a lower cost [8,9,10,11,12]. However, the elongation of commercial UNS S32101 was around 30%, which limited the application of lean DSSs in many fields [8,13,14].

Recently, some developed lean DSSs with martensitic transformation have been designed to bring transformation induced plasticity (TRIP) effect and improve the ductility of lean DSSs. Herrera has designed a novel Mn based ductile lean duplex stainless TRIP steel (Fe-19.9Cr-0.42Ni-0.16N-4.79Mn-0.11C-0.46Cu-0.35Si, wt%), which had 1 GPa ultimate tensile strength and an elongation to fracture of above 60% resulted from a sequential martensite transformation of γ → ε → α’ [14]. Choi has reported the effects of nitrogen addition on the strain-induced martensitic transformation of Fe-20Cr-5Mn-0.2Ni duplex stainless steel and found that the elongation was up to 60% in some lean DSSs containing 0.3% N [15]. In our work, a new lean duplex stainless steel with a composition of 20.53Cr-3.45Mn-2.08Ni-0.17N-0.31Mo has been developed. With a low Ni content, this resource-saving DSS can be less affected by price fluctuation of precious metal and reduce the risk of both production and uses. Additionally, the elongation has been improved to around 50% thanks to martensitic TRIP effect, which is obviously higher than that of 30% in S32101. Thus, this newly developed DSS holds a great potential to be the promising replacement of UNS S32101 in the advantage of improved properties.

In order to expand the application of this newly developed DSS used in this work, it is of great importance to obtain the suitable mechanical properties and good corrosion resistance. Solution heat treatment shows great impact to the mechanical and corrosion properties of DSS. The aim of the present work is to find the optimum solution heating temperature for the studied specimen based on investigating the relationship between the solution heating temperature and the corresponding mechanical properties and pitting corrosion resistance. The effect of annealing temperature on the microstructure, mechanical properties and corrosion behavior of this novel lean duplex stainless steel has been characterized by microscope, mechanical test and electrochemical technique.

## 2. Experimental Section

### 2.1. Materials

**Figure 1:**
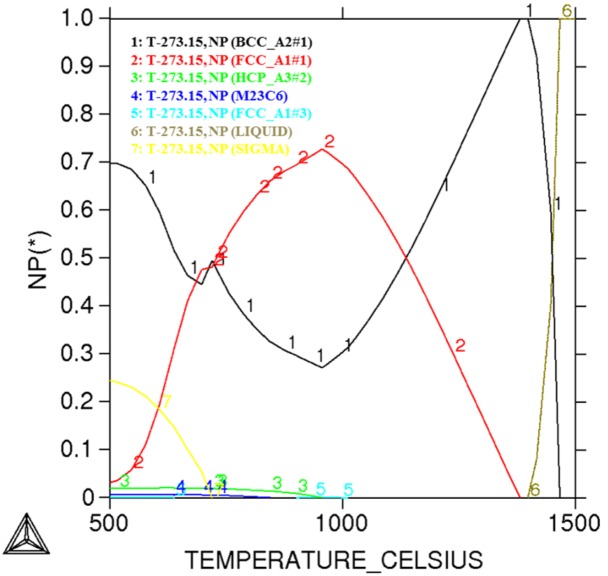
The phase diagram calculated by using the Thermo-Calc software for the newly developed DSS used in the present work.

The studied material in the paper was a novel developed lean duplex stainless steel with the chemical composition shown in Table 1. It was melted in a 50 kg vacuum furnace and then cast as a single square ingot. After removing the oxide skin, the ingot was forged into square slabs at the temperature ranging from 900 to 1200 °C with a thickness of 40 mm. The slabs were reheated at 1200 °C for 2 h and hot-rolled, using a laboratory hot-rolling mill, into 4 mm thick plates and then cold-rolled into 1.5 mm. The specimens were machined into blocks of dimension of 10 mm × 10 mm. The phase diagram of the newly developed DSS used in present work was calculated by using the Thermo-calc software, as shown in Figure 1. It could be seen that mainly ferrite and austenite were formed in the temperature range between 1000 and 1200 °C. Moreover, the specimen aged between 500 and 980 °C was subjected to precipitation of secondary phases, such as Cr_2_N, σ and secondary austenite (γ_2_), which would seriously deteriorate the corrosion resistance of the duplex stainless steel [16,17]. Therefore, in the present work, the solution heating temperature below 1000 °C was not chosen so as to avoid the formation of these detrimental secondary phases. Thus, the specimens were subjected to different annealing treatment at 1000, 1020, 1050, 1080, 1110, and 1150 °C for 30 min, respectively, and quenched in water to avoid intermetallic precipitates.

**Table 1:** Chemical composition (wt%) of the duplex stainless steels (DSS).

UNS No.	C	Si	Mn	P	S	Cr	Ni	Mo	Cu	N
S32750	≤0.03	≤0.8	≤1.2	≤0.035	≤0.02	24.0/26.0	6.0/8.0	3.0/5.0	≤0.5	0.24/0.32
S32101	<0.04	<1.0	4.0/6.0	<0.04	<0.03	21.0/22.0	1.35/1.70	0.10/0.80	0.10/0.80	0.20/0.25
New DSS	0.03	0.32	3.45	0.01	0.004	20.53	2.08	0.31	0.34	0.17

### 2.2. Uniaxial Tensile Test

The uniaxial tensile tests were conducted at room temperature with specimens having a gage thickness of 1.5 mm and width of 20 mm annealed at different temperatures using an Instron 5985 (Instron company, Pittsburgh, PA, USA) testing machine according to BS EN ISO 6892-1:2009 [18]. The test was carried out at an extension rate of 3 mm/min before reaching yield point and 20 mm/min after reaching yield point. The test was conducted with three parallel specimens for every annealing temperature and the average data were used for determination of the tensile strength, yield strength and elongation at break values in this paper. In order to observe the microstructure of annealed specimens, which were pre-stretched to 40%, the specimens were etched in 2% HF solution for 30 s, to make the ferrite, austenite, and martensite be distinguished in the optical images.

### 2.3. Electrochemical Measurement

Electrochemical corrosion behavior of the specimens were performed with a CHI 660D potentiostat (Shanghai Chenhua Instrument Co., Ltd, Shanghai, China). All the measurements were carried out with a three-electrode cell where a Pt foil acted as the counter electrode and a saturated calomel electrode (SCE) as the reference electrode. The specimens acting as working electrodes were sealed in epoxy resin. Prior to each experiment, the working electrode was wet mechanically ground, subsequently polished with a 1.5 μm diamond paste, ultrasonically cleaned in ethanol and distilled water, and then dried in air. To avoid the crevice corrosion, interfaces between specimen and resin were sealed with silica gel sealant, and dried in air. The exposed electrode surface area was 100 mm^2^. The test solution was 500 mL of l mol/L NaCl. According to ASTM G 150-99 (2010) [19], critical pitting temperature (CPT) determination of this novel lean duplex stainless steel was performed via potentiostatic measurements by applying a constant potential of 250 mV SCE and increasing the solution temperature at a rate of 1 °C/min from 2 °C. The CPT was defined as the temperature at which the current sharply increased to 100 µA. After testing, specimens were electrochemically etched for 8 s in 30 wt% KOH solution at applied voltage of 2 V, to make the ferrite and austenite be distinguished in the optical images, and then examined for pits [20]. The CPT measurements of the same specimen for every annealing temperature were repeated at least six times, and the typical data were chosen as the CPT values in this work.

### 2.4. Optical Metallographic Microscopy and SEM-EDS Analysis

Microstructural examination of specimens after the electrochemical measurements was conducted using optical metallographic microscopy and SEM (SCE Phillips XL30 FEG, Amsterdam, The Netherlands). The volume fraction of ferrite and austenite was evaluated by quantitative metallography based on the software attached to the microscope. Energy dispersive X-ray spectroscopy (EDS) was performed on the individual phases to obtain their elemental composition. Because of the insensitivity of EDS techniques to N, an approximate calculation was conducted to determine the N fraction in both phases. The level of N in ferrite phase was assumed to be the saturation value (around 0.05%) and that of austenite was calculated based on the content of N in the whole alloy and the phase volume fraction [21]. The SEM-EDS analysis was tested with six parallel specimens for every annealing temperature. Each value of the element distribution in Table 2 was an average of more than ten measurements.

**Table 2:** Chemical composition and pitting resistance equivalent number (PREN) of the two phases for specimens annealed at different temperatures, obtained by EDS.

Annealing temperature	Phase	Volume fraction (%)	Cr (%)	Ni (%)	Mo (%)	N (%)	PREN_16_	PREN_30_
1000 °C	Ferrite	52.1	21.59	1.51	0.40	0.05	23.710	24.410
	Austenite	47.9	19.37	2.70	0.21	0.30	24.863	29.063
1020 °C	Ferrite	57.9	21.45	1.68	0.32	0.05	23.306	24.006
	Austenite	42.1	19.27	2.62	0.29	0.34	25.667	30.427
1050 °C	Ferrite	61.4	21.24	1.72	0.33	0.05	23.129	23.829
	Austenite	38.6	19.40	2.66	0.28	0.36	26.084	31.124
1080 °C	Ferrite	62.9	21.25	1.52	0.32	0.05	23.106	23.806
	Austenite	37.1	19.31	3.03	0.30	0.37	26.220	31.400
1110 °C	Ferrite	66.2	21.15	1.73	0.35	0.05	23.105	23.805
	Austenite	33.8	19.32	2.77	0.22	0.41	26.606	32.346
1150 °C	Ferrite	69.1	21.05	1.85	0.32	0.05	22.906	23.606
	Austenite	30.9	19.37	2.60	0.29	0.44	27.367	33.527

### 2.5. Magnetic Force Microscopy (MFM) and Scanning Kelvin Probe Force Microscopy (SKPFM) Measurements

The MFM and SKPFM measurements were performed using a dimension Nanoscope V (Bruker Corporation, Santa Barbara, CA, USA). All measurements were conducted in air at room temperature and an ambient relative humidity of about 45%. The probes used in MFM measurements were Nanoprobe™ (Bruker Corporation, Santa Barbara, CA, USA) MSEP Co/Cr-coated tips with force constant of 2.8 N/m and resonant frequency of 75 kHz, while the probes in SKPFM measurements were SCM-PIT conductive Pt/Ir-coated tips with the same force constant and resonant frequency. A dual-scan mode was used to record a second signal in addition to surface topography signal in MFM and SKPFM measurements. The topography of the sample surface was obtained in the first scan with the tapping mode. In the second scan, the cantilever was lifted up 100 nm to avoid the influence of topographic features. The principle and details of the SKPFM measurement have been reported previously [22,23].

## 3. Results and Discussion

### 3.1. Microstructures and Elements Distribution

**Figure 2:**
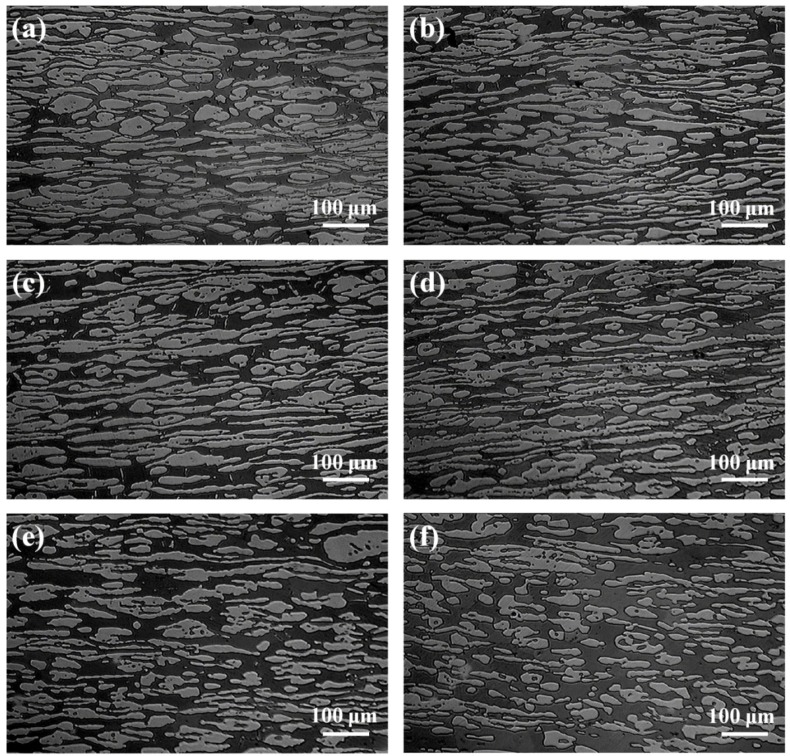
Optical micrographs of specimens annealed at different temperature for 30 min, the dark area representing ferrite while the light representing austenite. (**a**) 1000 °C; (**b**) 1020 °C; (**c**) 1050 °C; (**d**) 1080 °C; (**e**) 1110 °C; (**f**) 1150 °C.

**Figure 3:**
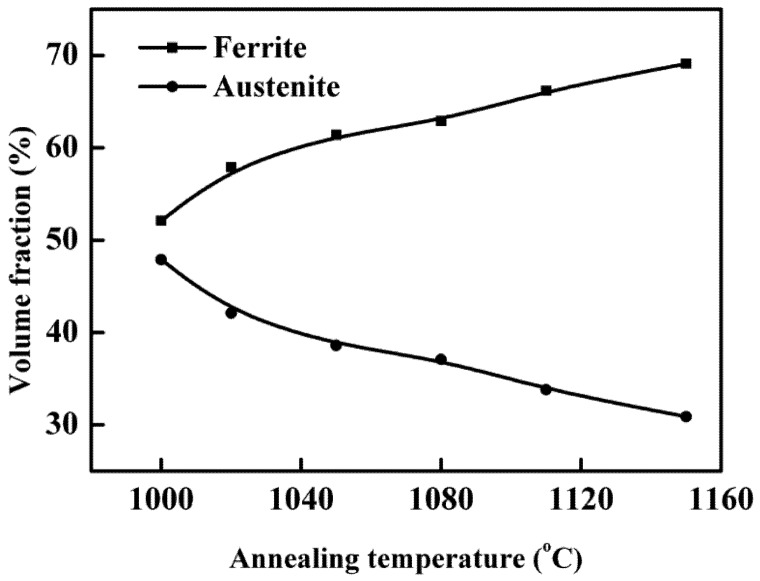
Volume fraction of ferrite and austenite phase with different annealing temperatures for 30 min.

Figure 2 showed the microstructure of specimens annealed at different temperatures for 30 min. The island-shaped austenite phase was evenly embedded in the band-shaped ferrite matrix. Figure 3 displayed the volume fraction of the ferrite/austenite phases against annealing temperature. It could be observed that as the annealing temperature ranged from 1000 to 1150 °C, the ferrite region gradually increased and austenite region decreased, which was a typical diffusion-controlled solid-state phase transformation: γ → α and determined by the equilibrium phase diagram [24]. Besides, the ferrite and austenite phase approximately reached balanced when annealed at 1000 °C (*i.e.*, Volume_Ferrite_:Volume_Austenite_ ≈ 1:1). However, Figure 1 revealed that the equal volume fraction of the two phases in the investigated specimens was achieved at annealing temperature higher than 1000 °C, which was attributed to the fact that the Thermo-Calc described thermodynamic equilibrium conditions while the steels could never be in true equilibrium [10]. The content of main alloying elements measured by EDX in austenite and ferrite phases was listed in Table 2. It was shown that alloying elements, such as Cr and Mo, were enriched in the ferrite phase, while N and Ni were concentrated in the austenite phase. As the annealing temperature was increased, content of Cr and Mo decreased in the ferrite phase, while the content of Cr and Mo in austenite increased slightly. It could be explained by that the higher annealing temperature, resulted in the higher volume fraction of ferrite, which further led that corresponding Cr and Mo were spread over a larger region and diluted [20].

### 3.2. Mechanical Properties

**Figure 4:**
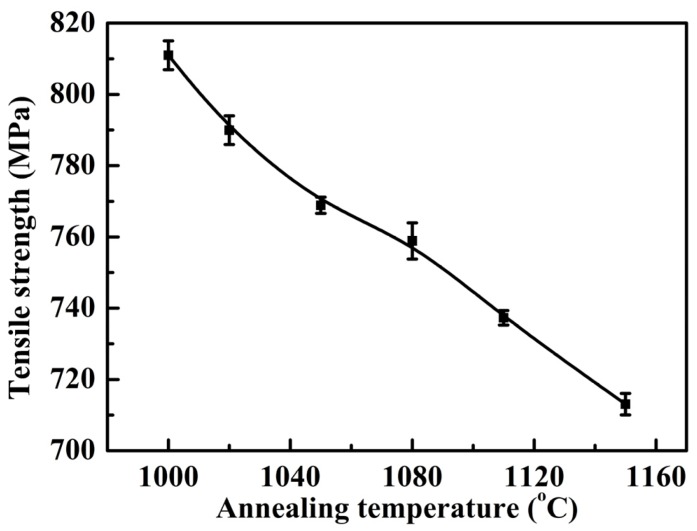
Tensile strength of specimens with different annealing temperatures for 30 min.

**Figure 5:**
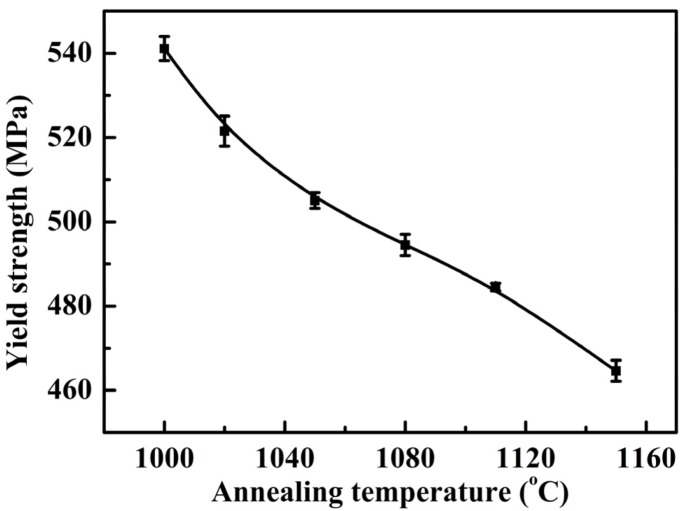
Yield strength of specimens with different annealing temperatures for 30 min.

**Figure 6:**
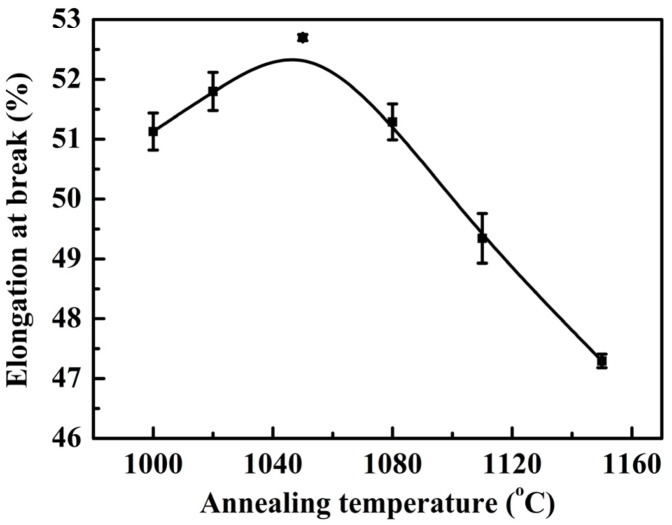
Elongation at break of specimens with different annealing temperatures for 30 min.

**Figure 7:**
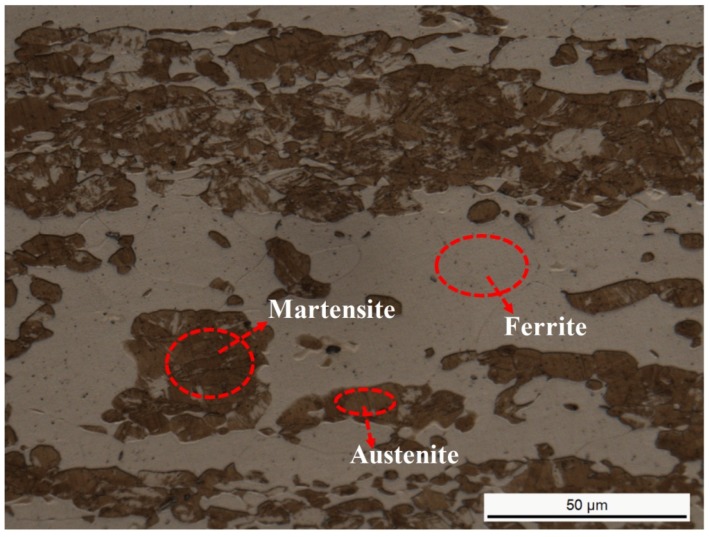
Optical micrographs of specimen annealed at 1050 °C for 30 min and pre-stretched to 40%, the dark area representing austenite while the light representing ferrite, and lath-shaped martensite was embedded in austenite phase.

Figure 4 and Figure 5, respectively, presented the tensile and yield strength of this novel lean duplex stainless steel as function of annealing temperature. The results showed that the tensile strength and yield strength decreased with increasing the annealing temperature from 1000 to 1150 °C. It was revealed that at 1000 °C, the specimens owned the highest tensile and yield strength, which could be attributed to the fact that the tensile and yield strength depended strongly on grain size [2,25,26]. According to the well-known Hall-Petch relation, a smaller average grain size resulted in a stronger material [27]. As the annealing temperature was increased from 1000 up to 1150 °C, the grain growth could be enhanced leading to the increased grain size, thus, the tensile and yield strength decreased. In addition, additional strengthening in DSSs could be caused by solid solution hardening [2]. The solid solution strengthening effects of chromium, molybdenum and nitrogen reached the maximum at 1000 °C as shown by the increased additions of alloying elements in Table 2. Figure 6 showed the elongation at break of this novel lean duplex stainless steel as function of annealing temperature. The elongation at break reached the maximum of 52.7% after annealing at 1050 °C and the minimum of 47.3% at annealing temperature of 1150 °C, being obviously higher than that of 25% in S32750 and 30% in S32101, which might be attributed to the martensite transformation [1,8,13,14]. Typical values of mechanical properties of solution annealed DSS were summarized in Table 3. The microstructure of specimen annealed at 1050 °C and pre-stretched to 40% is shown in Figure 7. It could be found that much lath-shaped martensite was embedded in dark retained austenite phase, which exhibited that austenite gradually transformed into martensite during tensile deformation. Moreover, it was already demonstrated that the strain-induced martensite transformation was helpful for improving the plasticity of the lean DSS associated with TRIP effect [15]. Meantime, it has been reported that the martensite transformation of lean DSS proceeded from austenite to hexagonal martensite to near cubic martensite (γ → ε → α’) [13,14]. However, Zhang has indicated that the main reason for the improvement of elongation of lean DSS was the γ → ε transformation, not in proportion to the generation of the α’ martensite [14]. For this novel lean DSS, the martensite transformation process remained to be investigated in detail. The elongation at break of specimen annealed at 1000 °C reached 51.13%, which was slightly lower than that of specimen after annealing at 1050 °C. However, combining the tensile and yield strength, the specimen annealed at 1000 °C demonstrated the best mechanical properties.

**Table 3:** Typical values of mechanical properties of solution annealed duplex stainless steels (DSS).

Alloy	Ultimate tensile strength, MPa	0.2% proof stress (min) MPa	Elongation (min) A5, %
UNS S32750 [1]	800–1000	550	25
UNS S32101 [8]	650–700	450	30
Newly Developed DSS	737–811	465	47.3

### 3.3. Pitting Corrosion Behavior

**Figure 8:**
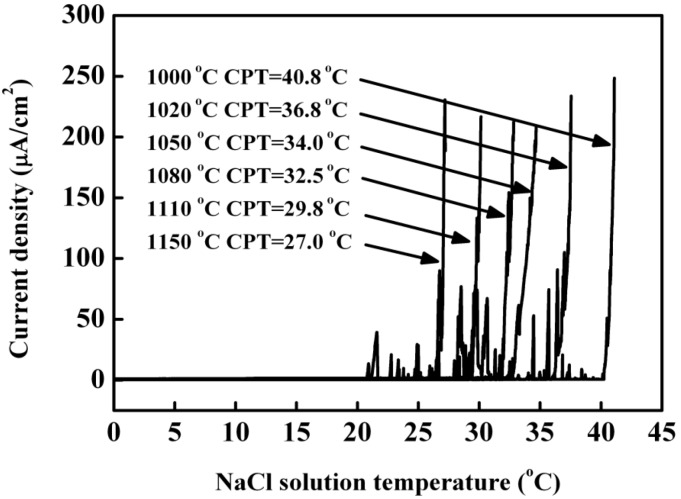
Curves of current density and temperature for specimens annealed at different temperatures in 1 M NaCl solution at an applied potential of 250 mV (SCE).

Figure 8 illustrated the typical curves of current density against annealing temperature for specimens annealed at different temperatures for 30 min. The current density presented a lower value less than 1 μA/cm^2^ during the initial heating, indicating that the steel surface was protected by the passive film. With the solution temperature increasing to CPT, some current density fluctuations were found, which were associated with a passive film breakdown in the form of metastable pits. As the temperature increased further reaching to the CPT, the current density rose sharply due to the occurrence of the stable pits. As the annealing temperature increased from 1000 to 1150 °C, the CPT decreased from 40.8 to 27 °C. The highest CPT, 40.8 °C, was obtained when the specimens were annealed at 1000 °C, which meant that the specimens annealed at 1000 °C showed the best pitting corrosion resistance. Compared with the lean duplex stainless steel UNS S32101, of which the highest CPT of 35.2 °C was obtained at annealing temperature of 1000 °C, this novel lean duplex stainless steel showed a better pitting corrosion resistance [10]. This could be explained by that this novel steel had higher content of N and lower content of Manganese (Mn) than S32101 [28].

**Figure 9:**
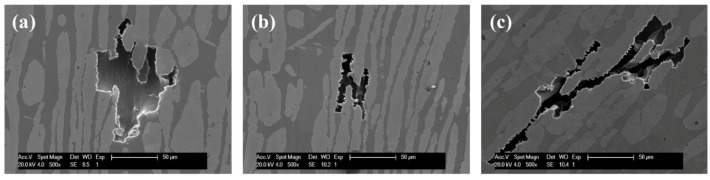
SEM morphologies of pittings formed on specimens annealed at different temperature for 30 min after the CPT test. (**a**) 1000 °C; (**b**) 1050 °C; (**c**) 1150 °C.

The morphologies of specimens were observed under SEM after electrochemical test and stable pits could be found. The stable pitting of specimens annealed at 1000 °C was located at both ferrite phase and austenite phase, while those of specimens annealed at temperatures over 1000 °C mainly distributed from both two phases to ferrite phase and the pitting corrosion occurred randomly. Especially for specimens annealed at 1150 °C, the stable pitting sites mainly attributed in the ferrite phase, and the pitting corrosion preferentially occurred in the ferrite phase. Typical stable pitting morphologies of the specimens annealed at 1000 °C, 1050 °C, and 1150 °C are shown in Figure 9.

The resistance to pitting corrosion in duplex stainless steels was found to be strongly dependent on the chemical composition. It was known that the pitting resistance equivalent number of single phase could be used to evaluate the corrosion resistance of duplex stainless steels quickly. The empirical formula of pitting resistance equivalent number was defined by the following equation:

PREN_16__–30_ = wt% Cr + 3.3 wt% Mo + 16–30 wt% N (1)

**Figure 10:**
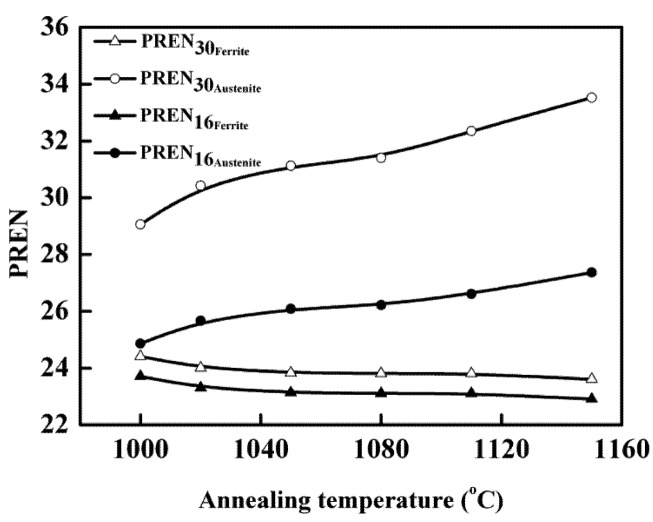
PREN for each phase of specimens with different annealing temperatures for 30 min.

Different subscript varying in this formula indicated the different nitrogen coefficient [28,29,30]. The alloy (or phase) with higher pitting resistance equivalent number would exhibit better pitting corrosion resistance. Both the PREN_16_ and PREN_30_ of ferrite and austenite were calculated and listed in Table 2. Based on this formula, as the annealing temperature increased, the pitting resistance equivalent number of ferrite phase decreased while that of austenite phase increased. Figure 10 showed the pitting resistance equivalent number of single phase as function of annealing temperature. Such results were caused by the change of chemical composition of each phase corresponding to the variation of annealing temperature [31,32,33]. It has been reported that the pitting resistance of duplex stainless steels was determined by the weaker phase not the entire alloy [7,34]. For this novel lean duplex stainless steel, when the specimens were annealed at the temperature 1000 °C, both PREN_16_ and PREN_30_ differences between austenite and ferrite reached the lowest, which meant the two phases had similar pitting resistance at 1000 °C. Therefore, the pitting was propagated at both phases, shown in Figure 9a. However, the difference of PREN_16_ between two phases was lower than that of PREN_30_, indicating that it was better to use PREN_16_ to explain the pitting corrosion behavior, which was in good agreement with the reported literature: 16 was the most frequently used subscript [2]. When the annealing temperature exceeded 1000 °C, the pitting resistance equivalent number of austenite phase was much higher than that of ferrite phase, and pitting mainly occurred in the ferrite phase. The pitting morphologies of specimen annealed at 1150 °C showed that pits were located mainly at ferrite areas, as shown in Figure 9c, indicating that the ferrite phase was much weaker than austenite phase. It could be found that the stable pitting morphologies of the specimens after CPT tests were fairly consistent with the pitting resistance equivalent number of the weaker phase for this new lean duplex stainless steel. It also could be obtained that the CPT reflected the pitting corrosion resistance of the weaker phase and that the pitting corrosion resistance of duplex stainless was determined by the weaker phase not the entire alloy.

### 3.4. MFM and SKPFM Results

**Figure 11:**
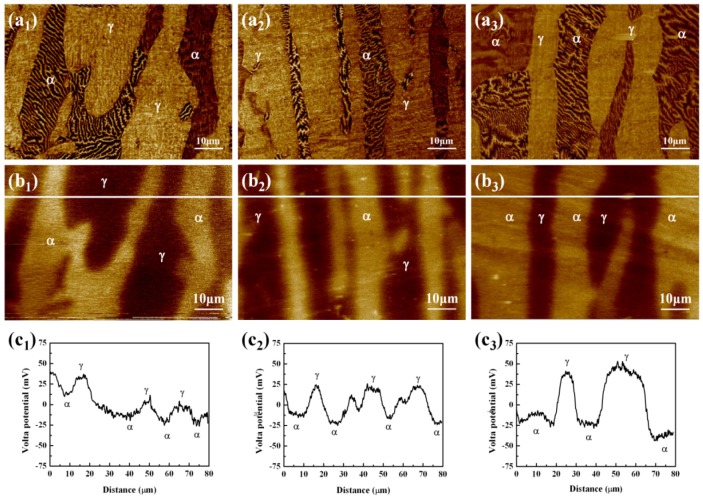
(**a_1_**–**a_3_**) MFM and (**b_1_**–**b_3_**) SKPFM images of specimens respectively annealed at 1000 °C, 1050 °C and 1150 °C for 30 min; (**c_1_**–**c_3_**) volta potential difference profile of a line as showed in (**b_1_**–**b_3_**).

Figure 11 showed the microstructure of specimens after annealing treatment at 1000, 1050 and 1150 °C for 30 min using MFM and SKPFM. Figure 11a_1_–a_3_ respectively displayed the MFM images of the specimens annealed at 1000, 1050 and 1150 °C with 80 μm × 80 μm, where striped ferromagnetic ferrite domain embedded in the paramagnetic austenite. Figure 11b_1_–b_3_ respectively revealed the SKPFM images of the same area with 80 μm × 80 μm. Figure 11c_1_–c_3_ demonstrated the Volta potential differences between the ferrite and austenite of specimens. The ferromagnetic ferrite had a lower Volta potential than the austenite. This was the same propensity as seen in the previous work [35,36,37]. The Volta potential difference between ferrite and austenite increased as the increase of annealing temperature. As shown in Table 4, the average potential difference between ferrite and austenite for the specimens annealed at 1000 °C, 1050 °C, 1150 °C were about 37.5 mV, 50 mV, and 75 mV. The highest value of Volta potential difference was obtained from specimen annealed at 1150 °C, and the lowest was from the specimen annealed at 1000 °C. A linear relationship between the Volta potential measured by SKPFM and corrosion potential has been reported [38,39,40,41,42]. It was reported that the possible galvanic interactions between the ferrite and austenite phases had been obtained by SKPFM technique [43]. In this paper, the ferrite had a lower Volta potential in this novel lean duplex stainless steel using SKPFM, which was in good agreement with the results in potentiostatic measurement (CPT) that pitting corrosion was found to be preferentially occurred in ferrite phase and the ferrite was a weaker phase. Compared with SKPFM technique, CPT measurement was performed at an applied potential. It couldn’t be explained by galvanic interactions. However, a conclusion might be obtained that the pitting corrosion tendency of ferrite increased when applying a static potential. In addition, the Volta potential difference between ferrite and austenite was well consistent with the difference between PREN_Ferrite_ and PREN_Austenite_, which could be explained by that the elements of Cr, Mo, and N should have certain influences on the Volta potential [35].

**Table 4:** The Volta potential difference between ferrite and austenite phase for the specimens annealed at 1000, 1050, and 1150°C.

Annealing temperature (°C)	Average Volta potential difference_Ferrite-Austenite_ (mV)
1000	37.5
1050	50
1150	75

## 4. Conclusions

A novel lean duplex stainless steel 20Cr-2Ni-3Mn-0.17N-0.31Mo has been developed with higher elongation at break and better pitting corrosion resistance than UNS S32101. It has a great potential to be the promising replacement of S32101 in the advantage of improved properties. In order to expand the application of this new developed duplex steel, it is of great importance to obtain the suitable mechanical properties and good corrosion resistance. Thus, the effect of annealing temperature on the microstructure, mechanical properties and corrosion behavior of this novel lean duplex stainless steel has been characterized by microscope, mechanical test and electrochemical technique. Based on the experimental results, it is discovered that the best mechanical properties and pitting corrosion resistance of this newly developed DSS can be obtained at annealing temperature 1000 °C, which should be of great help in the industrial production. The main conclusions are as follows.

(1) The volume fraction of austenite decreased continuously and the ferrite increased with the annealing temperature increasing from 1000 to 1150 °C.
(2) The tensile strength and yield strength decreased with the increase of annealing temperature, which could be explained by the grain size and solid solution strengthening effects of alloying elements.
(3) The elongation at break reached the maximum of 52.7% after annealing at 1050 °C due to martensite transformation associated with TRIPeffect.
(4) The critical pitting temperature decreased with increasing of annealing temperature. The localized pitting attack preferentially occurred at ferrite phase. The pitting corrosion behaviorin chloride solutions could be explained by redistribution of main alloying elements, such as chromium, molybdenum and nitrogen in two phases, resulting in the change of PREN_16_ of ferrite and austenite phases.
(5) Ferrite had a lower Volta potential than austenite phase. The Volta potential difference between ferrite and austenite increased as the increase of annealing temperature. The Volta potential difference between ferrite and austenite was in good conformity with the difference between PREN_Ferrite_ and PREN_Austenite_.
